# Compressive Yield Stress of Flocculated Kaolin Suspensions in Seawater

**DOI:** 10.3390/polym15030530

**Published:** 2023-01-19

**Authors:** Steven Nieto, Eder Piceros, Pedro G. Toledo, Pedro Robles, Ricardo Jeldres

**Affiliations:** 1 Departamento de Ingeniería Química y Procesos de Minerales, Facultad de Ingeniería, Universidad de Antofagasta, Antofagasta 1240000, Chile; 2 Faculty of Engineering and Architecture, Universidad Arturo Prat, Antofagasta 1240000, Chile; 3 Department of Chemical Engineering and Laboratory of Surface Analysis (ASIF), Universidad de Concepción, Concepción 4030000, Chile; 4 Escuela de Ingeniería Química, Pontificia Universidad de Valparaíso, Valparaíso 2340000, Chile

**Keywords:** seawater flocculation, kaolin clay, yield stress, compressive yield stress, mineral processing

## Abstract

The mining industry has resorted to using seawater while trying to find a solution to the water shortage, which is severe in some regions. Today, the industry looks to tailings dams to recover more water and, thus, increase recirculation. The migration of interstitial water due to the consolidation of particle networks can give rise to large water mirrors in different dam areas. These pools can contain enough water to be recovered and recirculated if the external stress caused by the weight of the pulp exceeds the compressive yield stress. The density and rheological properties of the discarded pulps determine the feasibility of water expulsion during tailings consolidation. As these conditions are largely established in the thickening stage, it is necessary to revisit operations, looking at the dam as a water source. Thus, a thorough understanding of the compressive properties that determine the level of consolidation of typical pulps and their relationships to aggregate properties, such as size and fractal dimension, is crucial. Here, the effect of two types of water, industrial water and synthetic seawater, on kaolin flocculation, sedimentation rate, yield stress, and compressive yield stress were studied. In addition, the relationship of these properties with the flocculant dose and the resulting aggregate size and fractal dimension was examined. One promising finding to practitioners was that salt and small doses of high molecular weight flocculant improved the consolidation of kaolin slurries under compression. These conditions generated low compressive yield stress compared to fresh water and water with low salt content, favoring the consolidation of the pulps and the release of water.

## 1. Introduction

The recovery of water during mineral processing is a crucial aspect of ensuring the sustainability of the mining industry, primarily when sulfide deposits are located in desert areas with little availability of water [1]. The primary operations to recover water are based on extraction from the flotation tailings in large thickeners where the particles settle, forming a concentrated sludge at the bottom. At the same time, an overflow of clarified water is generated, which is later reused in upstream operations. Efficiency improves after adding flocculants, which increases the particle settling rate and recovered water flow. Flocculants are high molecular weight, soluble polymers with functional groups that adhere to dispersed particles through various mechanisms, forming large agglomerates that settle quickly.

The thickened pulp is withdrawn through the lower cone of the thickener and then transported to the tailings storage facility (TSF). Depending on the density and rheological properties of the discarded pulp, it may be feasible to recover an additional amount of water expelled during consolidation [2,3]. The migration of interstitial water from the network of particles gives rise to large water mirrors in different dam areas. These pools can contain enough water to be recovered and recirculated if the external stress caused by the weight of the suspension exceeds the compressive yield stress, P_y_(ɸ), thus reordering the particles so that the solid material occupies a lower volume [4,5]. In this context, it is essential to understand the compressive properties’ behavior that determines the consolidation level of typical pulps, mainly when the tailings have been deposited in the TSF [6,7].

For the present study, it is particularly interesting to know the implications of the presence of clays and the use of seawater in the recovery of water, as these two factors are the most relevant challenges in the processing of sulfide minerals through concentration operations. Clays are fine minerals with a heterogeneous structure and a high surface area. Among the most common is kaolinite (Al_2_Si_2_O_5_(OH)_4_), which has a rigid, lamellar crystalline structure composed of silica/alumina bilayers with a spacing of 7.2 Å. The union formed by these sheets (octahedral/tetrahedral) is known as the 1:1 layer. This clay has two crystallographically different surfaces, the basal planes, or faces, and the edges. The faces have a permanent anionic charge in response to the isomorphic substitution of Al^3+^ by Si^4+^ in the tetrahedral silica and Mg^2+^ by Al^3+^ in the octahedral layer of the alumina [8]. The edges have a surface charge that varies (anionic or cationic) depending on the pH due to the protonation and deprotonation of the aluminol (Al-OH) and silanol (Si-OH) groups in the exposed planes with hydroxyl termination [9,10,11].

The high anisotropy of kaolinite leads to different modes of aggregation, including Edge-Edge (E-E), Edge-Face (E-F), and Face-Face (F-F) associations [12]. F-F associations form structured lamellar aggregates with a low volume and high density, known as card-pack flocs. These structures appear at a high pH (pH > 7) and/or high electrolyte concentrations and are characterized by low-yield stresses [13]. On the other hand, E-F and E-E associations form three-dimensional structures of a lower density and a higher apparent volumetric fraction, known as card-house flocs [14]. Generally, this type of structure occurs at a low pH (<6) and low electrolyte concentrations. It is characterized by a high compressive yield strength and a high apparent maximum packing fraction [15,16].

Seawater, meanwhile, contains a high ionic charge that modifies the nature of the interaction forces that govern the aggregation/dispersion phenomena between solid particles. For example, various studies have shown that anionic flocculants, based on polyacrylamides (PAM), roll up in a saline medium, limiting their ability to bridge particles [17,18]. This directly impairs the performance of the thickeners; however, the cations present in the liquid phase can generate additional bonds between the polymer and the mineral surface, improving the reagents’ adsorption. Recently, Nieto et al. [19] compared the aggregation phenomena of flocculated clayey tailings in freshwater and seawater. The largest aggregates were achieved in freshwater when the flocculant macromolecules were extended; however, a fraction of fine particles remained, resulting in a bimodal floc size distribution. On the contrary, smaller aggregates were generated in seawater when the flocculant chains were coiled. However, the fraction of single particles disappeared since the additional bonds due to cations, known as cationic bridges or salt bridges, allowed the primary particles to coagulate, thus facilitating polymer adsorption [20].

Nasser and James [15] analyzed the compressive yield stress, $P_{y}\left(\phi \right)$, of kaolinite suspensions, varying the concentration of sodium chloride and the pH. The kaolinite suspensions exhibited a power law dependence on the volume fraction of the kind $\;P_{y}\left(\phi \right)=c{\left(\phi \right)}^{n}$ in all cases. Next, Nasser and James [21] analyzed the effects caused by the physicochemical properties of flocculants on a kaolin suspension in freshwater. The compressive properties showed cationic PAM-based suspensions to be less compressible than anionic PAM-based suspensions. The electrostatic attraction between the positively charged cationic polymer and the negatively charged kaolinite allowed the cationic polymer to produce stronger flocs. In the case of anionic polymers, the electrostatic repulsion between the negatively charged polymer and negatively charged kaolinite surface caused the polymer molecules to be extended, leading to the formation of large, open, and fragile flocs. The structural characteristics of the particle aggregates defined the rheological behavior of a given suspension. For example, Jeldres et al. [22] related the microscopic properties of flocs, such as size and fractal dimension, with the yield stress of synthetic tailings flocculated with a high molecular weight anionic polymer in seawater. These authors found a monotonic relationship between the fractal dimension of the flocs and the yield stress of the pulp when the hydrodynamic conditions during flocculation were kept constant. Other reports in the literature showed a relationship between microscopic properties and macroscopic parameters of industrial interest, such as yield stress and sedimentation rates [23]. However, there are no systematic studies on the compressive properties of suspensions, which are vital for an industry that requires increasing the water circuit life cycle. This research deepens the study of kaolin flocculation to understand the differences caused by the water type, considering a low salinity system and a system with synthetic seawater. Particle sedimentation, shear yield stress, and compressive yield stress were measured and related, seeking a connection with aggregate properties, such as size distribution and fractal dimension.

The study was carried out under relevant conditions for the concentration circuits of the mining industry. The results present an optimistic scenario for the sustainability of mining in arid areas near the sea.

## 2. Materials and Methods

### 2.1. Materials

**Figure 1:**
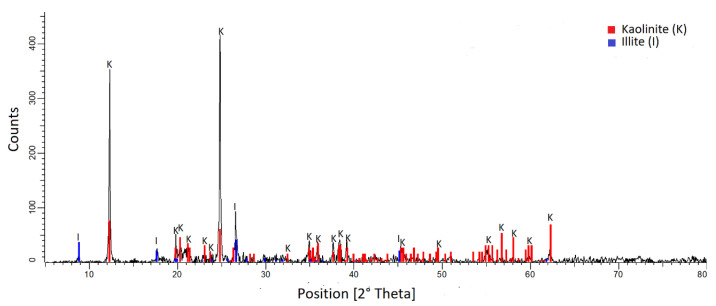
Kaolin X-ray diffractogram.

**Figure 2:**
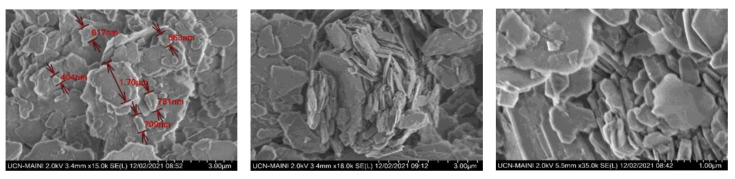
High-resolution SEM images showing the morphology of kaolin mineral.

Kaolin was purchased from Sigma-Aldrich (Burlington, MA, USA). The density was 2600 [kg/m^3^]. The mineral composition was determined by X-ray diffraction (XRD), using a Bruker X-ray diffractometer, model D8 advance. The wavelength λ(Cukα) was 1.5406 [Å], and the angle 2θ varied from 5° to 80°. The mineralogical components were analyzed using the Powder Diffraction File of ICDD (International Center for Diffraction Data). The diffractogram in Figure 1 confirms the majority presence of kaolinite and illite. The SEM micrograph (Figure 2) shows the presence of a population of submicron particles (<1 µm) with a hexagonal lamellar crystalline structure undetectable by the Focused Beam Reflectance Measurement (FBRM) probe.

The flocculant used was a commercial anionic polyacrylamide, SNF 704, provided by SNF Chile S.A. This reagent has a molecular weight of $18\times 10^{6}$ g/mol. Initially, a polymer stock solution was prepared in distilled water at a concentration of 1 g/L by continuous stirring for 24 h. Subsequently, an aliquot of the stock solution was diluted to 0.1 g/L. This solution was prepared daily and used in sedimentation, flocculation kinetics, and compressibility tests of flocculated pulps. The flocculant doses were expressed as grams of flocculant per ton of dry solid (g/t). The solutions used for the preparation of the kaolin suspensions were artificial seawater (SW) (Table 1) and industrial water (IW) (0.01 M NaCl). Distilled water was used for the preparation of both qualities of water. The salts and pH modifying reagent (NaOH) were of analytical grade.

### 2.2. Chemicals Analyses

The chemical analyses applied to SW, IW, and the supernatant phases of seawater-kaolin (K-SW) and industrial water-kaolin (K-IW) pulps were performed by atomic absorption spectrophotometry (Varian 220 FS Atomic Absorption Spectrophotometer, Varian, Palo Alto, CA, USA). The concentration of Na^+^, Ca^2+^, and Mg^2+^ ions in SW, IW, and the K-SW and K-IW extracts was measured. The concentration in the solution was determined by direct aspiration with an air-acetylene flame for the Na^+^ and Mg^2+^ ions and direct aspiration with a nitrous oxide-acetylene flame for the Ca^2+^ ion.

### 2.3. Flocculation-Sedimentation Tests and Size of Aggregates

**Figure 3:**
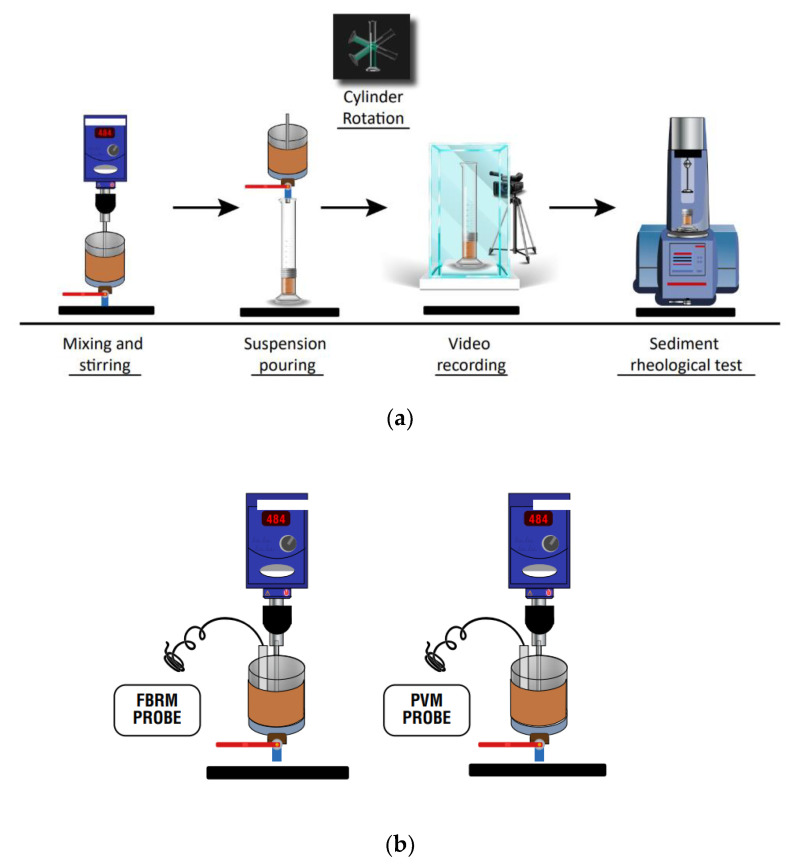
Schematic representation of the experimental setup for (**a**) sedimentation and sediment yield stress tests and (**b**) size characterization of aggregates with FBRM and PVM probes.

Kaolin slurries weight 300 g (5 wt%) were prepared in SW and IW. The suspensions were vigorously mixed for 20 min at 600 rpm using a 30 mm diameter Polytetrafluoroethylene (PTFE) turbine-type stirrer located in an axial position in a container (flocculation vessel) of a 100 mm diameter and 1 L capacity. The stirrer was located 20 ± 1 mm from the bottom of the vessel. Subsequently, the mixing speed was reduced to 180 rpm, and the flocculant was added at different dosages (50–150 g/t). The suspensions were prepared to carry out two types of tests. (a) Batch sedimentation tests (Figure 3a) were carried out after 15 s mixing of the flocculant and the suspension, gently pouring the suspension into removable cylinders (300 cm^3^ capacity and 35 mm internal diameter), and manually reversing the cylinders three times. After 1 h of sedimentation, the supernatant fluid was removed and homogenized. Then, a 20-mL aliquot was taken for turbidity measurements in a Hanna HI98713 turbidimeter that can do ten readings in 20 s, finally delivering the average of the readings. (b) For the characterization of aggregates (Figure 3b) using the FBRM technique, the probe was immersed vertically in the suspension, 10 mm above the PTFE stirrer, and 20 mm off-axis. The equipment used was a Particle Track G400 with FBRM technology from Mettler Toledo. The FBRM probe featured a laser beam focused through a sapphire window that scans a fast-circular path at a tangential speed of 2 m/s. When the laser beam encountered one of the particles, backscattered light was emitted that was proportional to the length of the intercepted particle, allowing size change and particle count to be tracked in real time. A Mettler-Toledo Particle Vision and Measurement (PVM) instrument (model V819) was used in the selected tests to capture real-time images of particles and aggregates. The same flocculation process was followed for the measurements with the FBRM probe. The PVM probe has a 19mm outer diameter tip and a sapphire window through which strobe light passes, allowing grayscale images (1075 × 825 µm) to be captured at 2 µm resolution.

### 2.4. Sediment Yield Stress

The yield stress measurements were made in an Anton Paar MCR 102 rheometer (ANAMIN Group, Santiago, Chile). The data were processed with the RheocompassTM Light version software (ANAMIN Group, Santiago, Chile). The paddle-in-cup configuration (model ST22-4V 40, Anton Paar, Graz, Austria) was used with the stress-strain method. The diameter of the cup was 4.2 cm, and that of the paddle was 2.2 cm. After one hour of sedimentation, the supernatant liquid was removed, and the lower part of the test tube containing the sediment was disassembled for rheological characterization (Figure 3a).

The yield stress was considered to be the average between the minimum stress necessary for the pulp to begin to flow and the stress that generated the maximum deformation (critical deformation). Up to the specified shear stress, the relationship between stress and strain was constant, representing a range of elastic strain. After this range, an irreversible deformation occurred, also called plastic deformation. The yield stress was detected in the logarithmic representation of the strain against the shear stress through the intersection of two tangent curves corresponding to the elastic and plastic regions of the sediment. Measurements were made in a logarithmic ramp, considering an initial duration of 60 s and a final duration of 1 s, increasing the shear stress value with an interval of 1 Pa.

### 2.5. Fractal Dimension

The fractal dimension, $D_{f}$, is a parameter that provides valuable information about the structure of the aggregates. $D_{f}$ can take values between 1–3, considering one as a one-dimensional line and three as a solid sphere. $D_{f}$ was obtained from the model proposed by Heath et al. [24] that related the diameter of the aggregates with the hindered sedimentation rate from sedimentation tests (Equation (1)). For the diameter of the aggregates, the squared weighted mean chord length obtained by the FBRM technique was used.

(1) $$U_{h}=\frac{d_{agg}^{2}g\left(\rho_{s}-\rho_{l}\right){\left(\frac{d_{agg}}{d_{p}}\right)}^{D_{f}-3}}{18\mu }{\left(1-\varphi_{s}{\left(\frac{d_{agg}}{d_{p}}\right)}^{3-D_{f}}\right)}^{4.65}$$

where, $U_{h}$ is the hindered sedimentation rate [m/s]; $d_{agg}$ and $d_{p}$ are the aggregate and particle diameters [m], respectively, which approximate the weighted mean squared chord length obtained with the FBRM probe; g is the acceleration due to gravity, 9.81 m/s^2^; $\rho_{s}$ and $\rho_{l}\;$ correspond to the density of the solid (2600 kg/m^3^) and liquid (IW: 1007 kg/m^3^ and SW: 1025 kg/m^3^), respectively; μ is the viscosity of the fluid (IW: 0.001021 Ns/m^2^ and SW: 0.001077 Ns/m^2^); $\varphi_{s}\;$ is the volumetric fraction of solids ≈ 0.02 *v/v*; and $D_{f}$ is the fractal dimension.

### 2.6. Compressive Yield Stress

**Figure 4:**
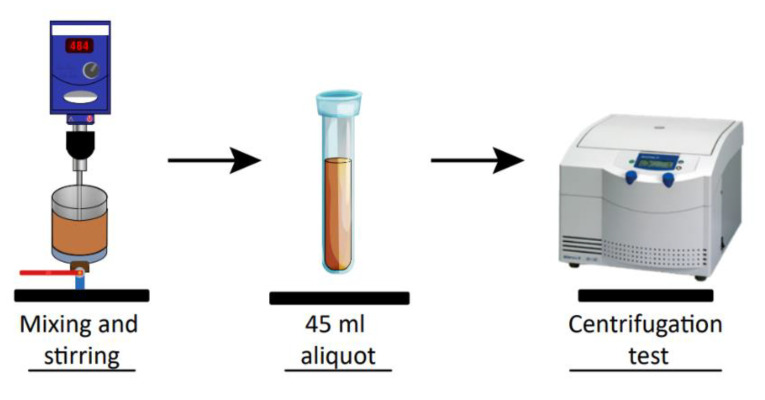
Schematic representation of the experimental setup for the centrifugation assays.

For the characterization of the compressive yield stress, several authors have determined the compressive yield strength $P_{y}\left(\phi \right)$, of various suspensions, such as cemented pastes [7], kaolinite [15,25,26], and latex and bentonite [5] using batch centrifugation. Here kaolin suspensions were prepared by varying the flocculant dosage (50–100 g/t) and water quality (SW and IW) following Section 2.3. Tests for $P_{y}\left(\phi \right)$ were performed on a representative 45 mL sample of each suspension in a Sigma Model 26E centrifuge with a 4-position swing-out rotor and 4 × 50-mL tube wells. Each suspension was subjected to increasing centrifugal speeds to obtain curves of equilibrium sediment height, $H_{eq}$, versus centrifugal speed, $\vec{g}$, data that were then fitted as shown in Equation (2) with parameters *a*, *b*, and *c*. The fraction of solids by volume ϕ for each centrifugal speed was obtained from Equation (3) with $\phi_{0}$, the initial volume fraction of solids, $h_{0}$, the initial height of the suspension, and *R,* the radius from the center of the centrifuge to the base of the test tube. $P_{y}\left(\phi \right)$ was then calculated from Equation (4) for each selected level of centrifugal acceleration (200–2466 $\vec{g}$) using the method described by Green [27]. Figure 4 shows a schematic representation of the experimental setup for the centrifugation assays.

(2) $$H_{eq}=a+b\ln\vec{g}+c\ln{\left(\vec{g}\right)}^{2}+d\ln{\left(\vec{g}\right)}^{3}$$

(3) $$\phi =\frac{\phi_{0}h_{0}\left[1-\frac{1}{2R}\left(H_{eq}+\vec{g}\frac{dH_{eq}}{d\vec{g}}\right)\right]}{\left[\left(H_{eq}+\vec{g}\frac{dH_{eq}}{d\vec{g}}\right)\left(1-\frac{H_{eq}}{R}\right)+\frac{H_{eq}^{2}}{2R}\right]}$$

(4) $$P_{y}\left(\phi \right)=\phi \rho \phi_{0}h_{0}\vec{g}\left(1-\frac{H_{eq}}{2R}\right)$$

where ∆*ρ* is the difference between the solid and liquid densities.

## 3. Results and Discussion

### 3.1. Chemical Analyses

Table 2 shows the chemical analyzes of IW, SW, and supernatant water of the kaolin suspensions prepared with the two types of water at pH 7 (K-IW and K-SW suspensions). The results indicate that the presence of kaolin in IW slightly increased the dissolved ions of Ca^2+^, Mg^2+^, and Na+, which can be attributed to contaminants. On the other hand, the supernatant water of K-SW suspensions reduced Mg^2+^ and Na^+^ and increased Ca^2+^, likely in response to ionic exchange between kaolin and SW.

### 3.2. Initial Settling Rate

**Figure 5:**
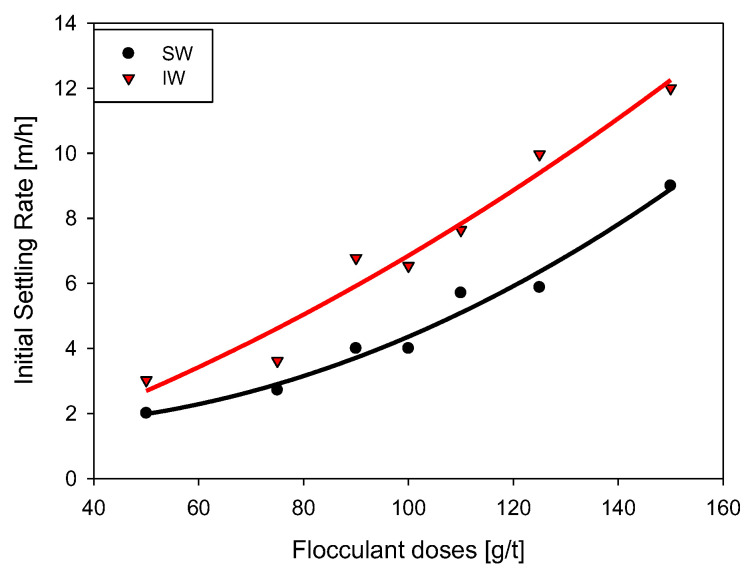
Effect of flocculant dosage on the initial sedimentation rate of kaolin in SW and IW. Stirring rate 180 rpm, solids concentration 5 wt%, flocculation time 15 s, and pH 7.

The initial sedimentation rate is a relevant measure in thickening operations. It is a great help to understand how thickener efficiency is affected by operating variables such as flocculant dosage, pH, water quality, and solids concentration, among others. Figure 5 shows that the sedimentation rate increases monotonically with the increase in the flocculant dose (50–150 g/t). The highest rates were obtained for kaolin suspensions in IW, with values between 3–12 m/h, while in SW, values between 2–9 m/h were obtained. As reported by Jeldres et al. [28], the coiling of the flocculant chains increased with the concentration of salts in the solution, limiting the ability of polymer bridges to form larger aggregates. According to recent results by Quezada et al. [20], the adsorption of HPAM on the kaolin surface occurred through the nitrogen of the polymer with the deprotonated oxygen of the kaolin. However, in a saline medium, cationic bridges were predominant, favoring the interactions of the deprotonated oxygen of the acrylic group of the HPAM with the deprotonated oxygen of the kaolin surface.

### 3.3. Flocculation Kinetics

**Figure 6:**
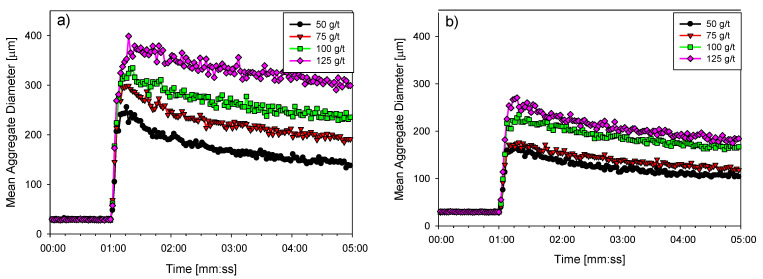
Effect of the flocculant dose on the evolution of the average size of kaolin aggregates in IW (**a**) and SW (**b**). Mixing rate 180 rpm, solids concentration 5 wt%, and pH 7.

In this section, the kinetic profiles obtained from the FBRM technique for kaolin suspensions flocculated with different doses of high molecular weight anionic polyacrylamide in IW (Figure 6a) and SW (Figure 6b) are analyzed. Initially, the average chord length was about 25 µm. At minute one, the flocculant was added; the size of the aggregates increased rapidly until reaching a maximum value a few seconds after the start of flocculation. This maximum strongly depended on the dose of polymeric reagent. For kaolin suspensions in IW, values between 225–380 µm were obtained with doses between 50–125 g/t. However, these values were slightly lower in SW, moving into the 170–270 µm range for the same dose range due to flocculant coiling that limited the ability to form polymeric bridges.

### 3.4. Chord Length Distributions (CLD)

**Figure 7:**
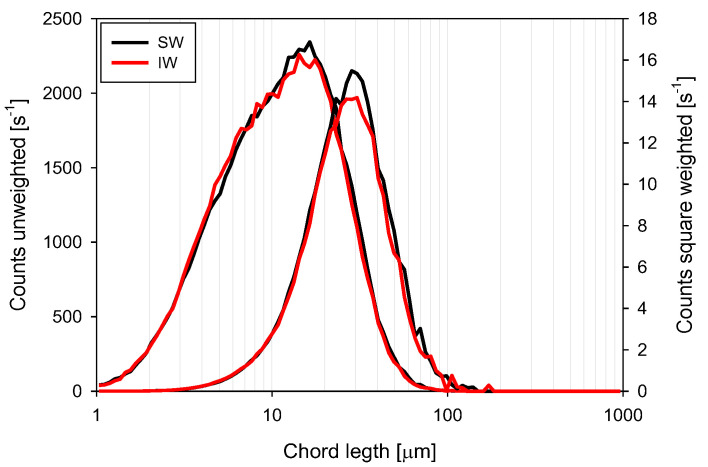
Unweighted and square-weighted chord length distribution from non-flocculated of kaolin suspensions in IW and SW. Solid concentration 5 wt% and pH 7.

Non-flocculated CLD: Figure 7 shows the unweighted and square-weighted chord length distributions of kaolin particles in the presence of IW and SW. The unweighted distribution offers information in the fine particle region, while the square-weighted distribution is more sensitive to larger particles, favoring the perception of changes in the coarse particle region [29]. An increase in the ionic strength of the system favors particle coagulation in response to the presence of cations such as Na^+^, Ca^2+^, and Mg^2+^, which reduce the length of the electrical double layer that surrounds the particle surface [30,31]. Thus, a change in water quality from IW to SW led to an almost imperceptible shift of the CLD towards larger aggregate sizes and a clear increase in the maximum height of the dominant size peak.

**Figure 8:**
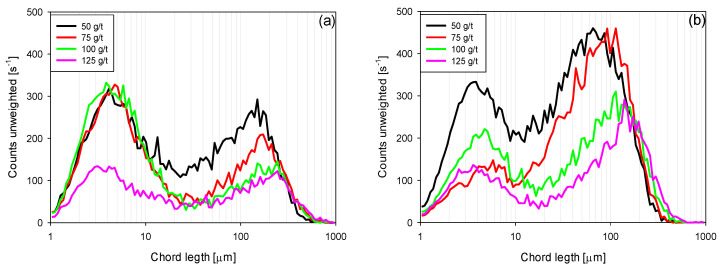

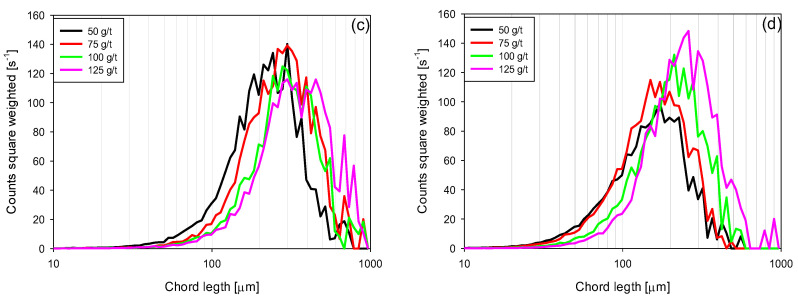
Unweighted (**a**,**b**) and square-weighted (**c**,**d**) chord length distribution from flocculated kaolin suspensions in IW (**a**,**c**) and SW (**b**,**d**) for different flocculant doses at 180 rpm. Solid concentration 5 wt%, reaction time 15 s, and pH 7.

Flocculated CLD: Figure 8 shows the unweighted and square-weighted chord length distributions for IW and SW flocculated kaolin suspensions. As the flocculant dose increased, particle aggregation increased, consequently decreasing the total number of particles. In both types of water, a fraction of particles remained non-flocculated, which is suggested by the bimodal shape of the unweighted distributions (Figure 8a,b), where the first peak represents the finer particles not bridged by the flocculant (3–6 µm). The second peak corresponds to the largest aggregates generated by polymer bridges. As expected, the amount of non-flocculated particles decreased with increasing reagent dosage. For example, in IW, with a dose of 50 g/t, a maximum height of the first peak of counts at 320 s^−1^ was obtained, which decreased to 120 s^−1^ with a dose of 125 g/t. In SW, the peak occurred at 335 s^−1^ at 50 g/t and dropped to 135 s^−1^ at 125 g/t. However, in both types of water, the coarse aggregates increased in size with increasing flocculant dosage, as best represented by the square-weighted chord distribution (Figure 8c,d), with larger aggregates appearing in IW, especially with the higher doses of polymer. The portion of fine particles that remained non-flocculated appears significant (Figure 8a,b); however, as they had low mass, the bimodal distribution was not perceived in the square-weighting modes (Figure 8c,d).

### 3.5. Yield Stress

**Figure 9:**
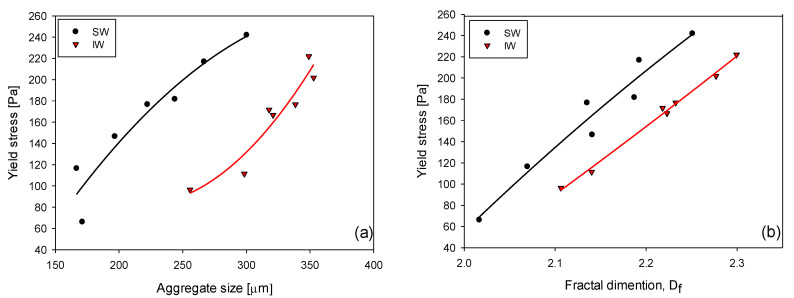
Relationship between sediment yield stress and (**a**) aggregate size (mean chord length) and (**b**) fractal dimension in IW and SW. Shear rate 180 rpm, solid concentration 5 wt%, flocculation time 15 s, and pH 7.

Next, the rheological analysis of the sediments obtained after one hour of sedimentation of pulps that have been flocculated for 15 s is presented. The yield stress results strongly depended on the microscopic properties of the aggregates, such as aggregate size and fractal dimension. Figure 9a shows that the yield stress increased significantly with the size of the aggregates, which in turn depended not only on the flocculant dose and flocculation time but also on the type of water. Thus, for the same size of aggregates, the yield stress was much higher in SW than in IW, revealing more resistant aggregates in SW and, most likely, with a more compact structure. Figure 9b shows that the yield stress increased significantly with the fractal dimension of the aggregates, which also depended on the flocculant dose, flocculation time, and the type of water. These results revealed that higher doses of flocculant in a saline medium obtain more compact aggregates with greater fractal dimensions, as expected. This result agrees with a previous study [22], in which it was established that at a fixed mixing rate, the yield stress followed an exponential relationship with the fractal dimension. This also agrees with Deng and Davè [32]. Using the model proposed by Kendall and Stainton [33], they established that the decrease in the mechanical resistance of aggregates is strongly related to low fractal dimensions.

**Figure 10:**
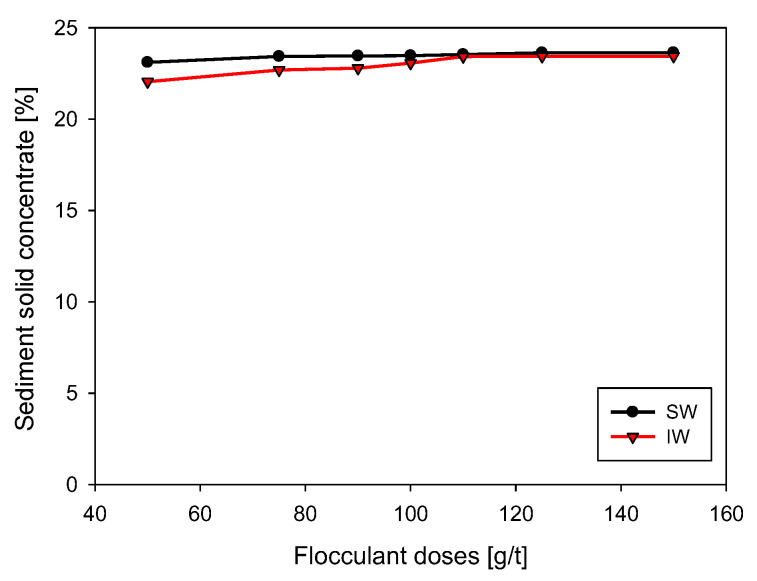
Effect of flocculant dosage on solids concentration in kaolin sediment in IW and SW. Stirring rate 180 rpm, solids concentration 5 wt%, flocculation time 15 s, and pH 7.

The increase in flocculant dose also contributed to the increase in rheological parameters in at least two ways. (i) A greater number of bonds between particles that increase the strength of the particle network and those that make up the suspension and (ii) a higher percentage of solids in the sediment (Figure 10), which was higher for SW (23.1–23.6 wt%) compared to IW (22.1–23.4 wt%).

### 3.6. Compressive Yield Stress

Here, the relationship between the compressive yield stress ($P_{y}\left(\phi \right)$) and the volumetric fraction of the kaolin sediment (ɸ) with different flocculant doses in industrial water (IW) and seawater (SW) is presented. Suspensions were prepared according to Section 2.5.

**Figure 11:**
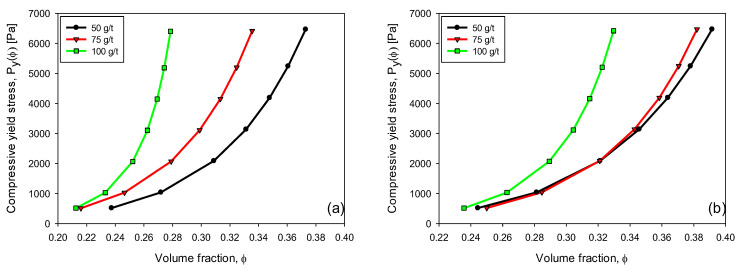
Variation of compressive yield stress versus volume fraction of kaolin suspensions with flocculant dose in IW (**a**) and SW (**b**). Shear rate 180 rpm, solid concentration 5 wt%, flocculation time 15 s, and pH 7.

Compressive rheology can be characterized through the compressive yield stress, which describes and characterizes the behavior of suspensions during their consolidation. $P_{y}\left(\phi \right)$ provides information on the resistance of the sediment when some external force compresses it. A higher value of $P_{y}\left(\phi \right)$ means that the sediment is less likely to compress and release water from within. Figure 11 shows that $P_{y}\left(\phi \right)$ strongly depended on the flocculant dose and water type. Adding a flocculant in increasing doses, from 50 to 100 g/t, led to increasing $P_{y}\left(\phi \right)$ by several orders and more rapidly in IW than in SW. Leong [34] considered the effect of polyacrylic acid (PAA) of low and high molecular weights (2000 and 750,000 g/mol, respectively) on the compression behavior of ZrO_2_ suspensions, with an initial volumetric solids concentration of 0.159. Low molecular weight PAA coated the zirconia particles, generating a steric repulsion that improved suspension consolidation under compression compared to the sediment without PAA. On the other hand, adding high molecular weight PAA did not improve the consolidation of solids under compression because the long polymeric chains increased the strength of the particle network (in addition to van der Waals forces). This last case closely captures the consolidation behavior of kaolin suspensions as the flocculant dose increases (Figure 11). Miller et al. [7] investigated the behavior of $P_{y}\left(\phi \right)$ in alumina suspensions by varying the degree of flocculation by changing the pH. This occurred at pH 4 with weak aggregation and at pH 9 with strong aggregation. In this case, $P_{y}\left(\phi \right)$ was higher for the strongest particle network by several orders of magnitude, replicating the consolidation behavior of high-dose flocculant kaolin suspensions.

**Figure 12:**
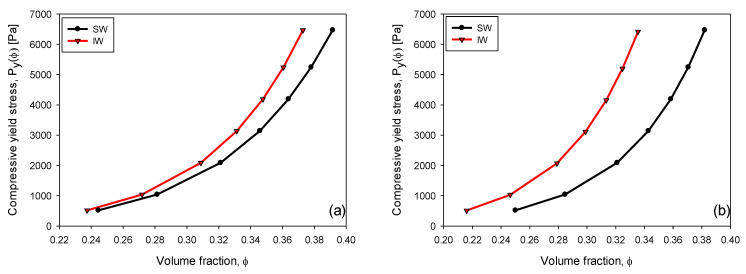

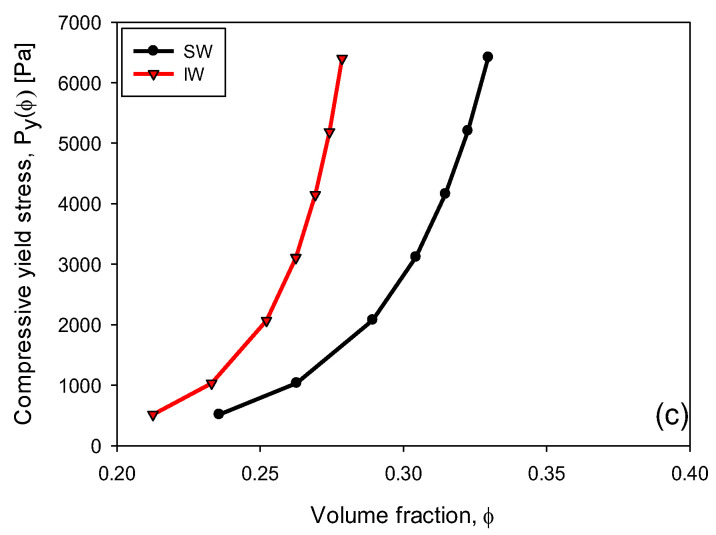
Variation of compressive yield stress versus volume fraction of kaolin suspensions in IW and SW with flocculant doses (**a**) 50 g/t, (**b**) 75 g/t, and (**c**) 100 g/t. Shear rate 180 rpm, solid concentration 5 wt%, flocculation time 15 s, and pH 7.

Figure 12 compares the compressive yield stress relationship versus volumetric fraction of kaolin sediments in the presence of IW and SW for three doses of flocculant (50, 75, and 100 g/t). The results suggest a power law of the form $P_{y}\left(\phi \right)=c{\left(\phi \right)}^{n}$, where $P_{y}\left(\phi \right)$ is the compressive yield stress, ɸ is the volumetric fraction of solids, *c* is a constant prefactor that amplifies the value of $P_{y}\left(\phi \right)$, and *n* is a constant representing the shape of the curve (the higher *n* the steeper the increase in $P_{y}\left(\phi \right))$. The results of *c* and *n* for the flocculated kaolin suspensions at each of the three doses of flocculant are presented in Table 3. Kaolin suspensions in IW showed higher compressive yield stress and lower sediment volume fractions than in SW. The parameters *c* and *n* in Table 3 always take the highest values in IW. A similar behavior was obtained by Nasser and James [15], who analyzed the consolidation of kaolinite suspensions by varying the concentration of NaCl (0.001–1 M) through centrifugation tests. These authors found that for the same volumetric fraction of solids at pH 7, $P_{y}\left(\phi \right)$ decreased with NaCl concentration. At the same time, at any given $P_{y}\left(\phi \right)$, the volumetric fraction of solids was higher in media with high salt. These authors related high values of $P_{y}\left(\phi \right)$ (low salt or freshwater) with bulky, three-dimensional, low-density E-F structures [35,36], and low values of $P_{y}\left(\phi \right)$ (high salt) with compact, high-density F-F structures. In a different system, Franks et al. [4] studied the consolidation of alumina suspensions, finding that $P_{y}\left(\phi \right)$ followed the polymer > high salt > low salt trend, which, at least with respect to the effect of salt, is different. Our results follow the consolidation trend of Nasser and James [15]. In our case, the pH is 7; therefore, the existence of E-F structures in IW was only marginal but sufficient to generate higher values of $P_{y}\left(\phi \right)$ than in SW, which favors F-F structures. This difference becomes pronounced as the flocculant dose increases.

### 3.7. Aggregate Images

**Figure 13:**
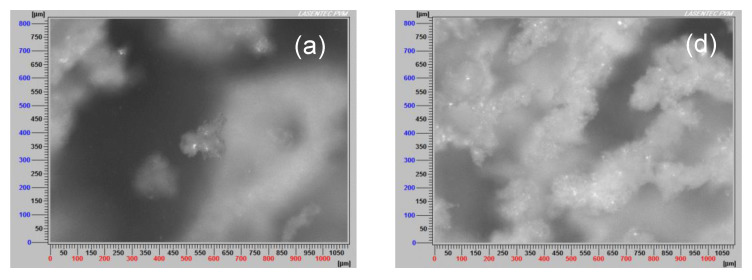

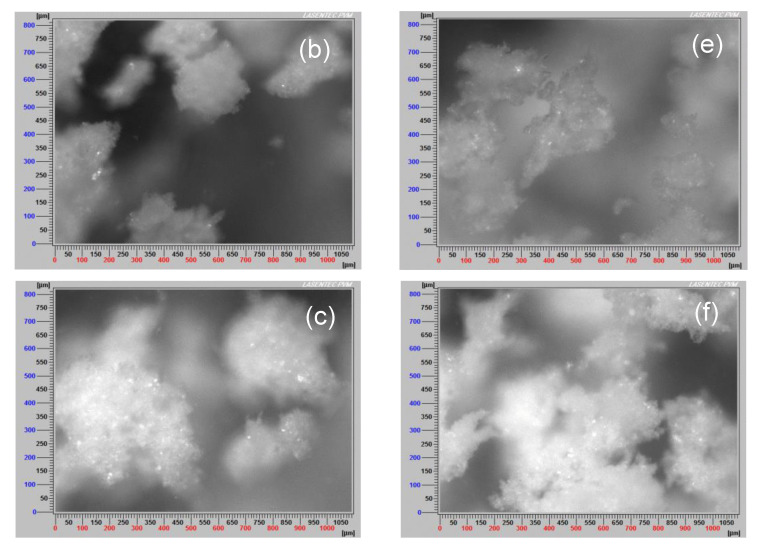
PVM images of flocculated kaolin suspensions with solid concentration 5 wt% and different doses of flocculant (50, 75, and 100 g/t) in IW (Frames a, b, c respectively) and SW (Frames d, e, f respectively) for a flocculation time of 15 s, fixed mixing rate of 180 rpm, and pH 7.

It was not easy to capture meaningful images of aggregates in kaolin suspensions. Figure 13 shows PVM images of flocculated kaolin suspensions with different doses of flocculant (50, 75, and 100 g/t) in IW (Figure 13a–c) and SW (Figure 13d–f) for a flocculation time of 15 s (approximate) and a fixed mixing rate of 180 rpm. At a low flocculant dose (50 g/t), larger and denser aggregates were seen in SW compared to IW. This is because, in SW, small flocs are interconnected from one to the other in an extended network. As the flocculant dose increased (up to 100 g/t), the aggregates grew, and the particle network spread further and became more intricate in SW. However, large aggregates also appeared in IW, although not interconnected. These findings correlate well with the size results of FBRM (Figure 6).

## 4. Conclusions

Water quality affects flocculation, sedimentation, yield stress, and consolidation of kaolin suspensions flocculated with a high molecular weight anionic polyelectrolyte. The present study led to the following conclusions.

The sedimentation rate strongly depends on the salinity of the suspension. The best performance was obtained in industrial water, with values between 3–12 m/h, where polymer stretching generates relatively large aggregates. The high salinity of seawater limits the ability of the flocculant to form polymeric bridges with the surface of the particles, generating a greater number of aggregates, although somewhat smaller and with more compact structures.

The chord length distribution of the aggregates is bimodal, with a mode corresponding to fine particles that remain unflocculated and a mode corresponding to large agglomerates generated by the action of the flocculant. A higher reagent dose and highly saline water presence depress the non-flocculating particle mode.

The yield stress of the sediment depends on the properties of the flocs that are generated prior to sedimentation. In this sense, the growth and compaction of the flocs produce high-yield stress values of the sediment. For example, in SW, with an aggregate size of 300 µm, a yield stress of approximately 242 Pa was obtained, while in IW, the yield stress was approximately 132 Pa.

Using high molecular weight flocculants such as anionic PAM makes it difficult to consolidate kaolin. An increase in the flocculant dose increases the compressive yield stress by several orders of magnitude. In addition, the increase in salinity causes a rearrangement of the aggregation of the primary particles, favoring F-F structures at pH 7.

For applications, salt and small doses of high molecular weight flocculant improve suspension consolidation under compression by generating low compressive yield stress values relative to freshwater or low salt water.

## Figures and Tables

**Table 1:** Chemical composition of seawater (SW) used.

Salt	Concentration [g/L]
NaCl	24.53
MgCl_2_·6H_2_O	11.10
Na_2_SO_4_	4.09
CaCl_2_	1.16
KCl	0.69
NaHCO_3_	0.20
KBr	0.10
H_3_BO_3_	0.03

**Table 2:** Dissolved salts in SW, IW, and supernatant water in suspensions of kaolin in seawater (K-SW) and industrial water (K-IW).

	[Mg^2+^] _Total_	[Ca^2+^] _Total_	[Na^+^] _Total_
SW	1338	339	11,130
IW	0	0	232
K-SW	1305	396	10,840
K-IW	0.8	1.1	254

**Table 3:** Power law parameters (*c* and *n*) and goodness of fit (*R*^2^) for the compressive yield stress of kaolin suspensions with three flocculant doses in SW and IW.

Water	50 g/t			75 g/t			100 g/t		
	$c\left(\times 10^{6}\right)Pa$	n	$R^{2}$	$c\left(\times 10^{6}\right)Pa$	n	$R^{2}$	$c\left(\times 10^{6}\right)Pa$	n	$R^{2}$
SW	0.95	5.36	0.9987	2	5.95	0.9977	20	7.50	0.994
IW	2	5.59	0.9985	3	5.73	0.9986	800	9.28	0.9883

## Data Availability

The data presented in this study are available on request from the corresponding author.
